# Patient preferences on rheumatoid arthritis second-line treatment: a discrete choice experiment of Swedish patients

**DOI:** 10.1186/s13075-020-02391-w

**Published:** 2020-12-19

**Authors:** Karin Schölin Bywall, Ulrik Kihlbom, Mats Hansson, Marie Falahee, Karim Raza, Eva Baecklund, Jorien Veldwijk

**Affiliations:** 1 Centre for Research Ethics & Bioethics, Department of Public Health and Caring Sciences, Uppsala University, Husargatan 3, Box 564, 752 37 Uppsala, Sweden; 2 Institute of Inflammation and Ageing, University of Birmingham Research Laboratories, Queen Elizabeth Hospital, Birmingham, B15 2WB UK; 3 Sandwell and West Birmingham Hospitals NHS Trust, Birmingham, B18 7QH UK; 4 Department of Medical Sciences, Rheumatology, Uppsala University, SE-751 85 Uppsala, Sweden; 5 Erasmus School of Health Policy & Management, and Erasmus Choice Modelling Centre, Erasmus University Rotterdam, P.O. Box 1738, 3000 DR Rotterdam, The Netherlands

**Keywords:** Discrete choice experiment, Patient preferences, Rheumatoid arthritis, Second-line treatment

## Abstract

**Background:**

Preference assessments of patients with rheumatoid arthritis can support clinical therapeutic decisions for including biologic and targeted synthetic medicines to use. This study assesses patient preferences for attributes of second-line therapies and heterogeneity within these preferences to estimate the relative importance of treatment characteristics and to calculate the minimum benefit levels patients require to accept higher levels of side effects.

**Methods:**

Between November 2018 to August 2019, patients with rheumatoid arthritis were recruited to a survey containing demographic and disease-related questions as well as a discrete choice experiment to measure their preferences for second-line therapies using biologics or Janus kinases inhibitors. Treatment characteristics included were route of administration, frequency of use, probability of mild short-term side effects, probability of side effects changing appearance, probability of psychological side effects, probability of severe side effects and effectiveness of treatment.

**Results:**

A total of 358 patients were included in the analysis. A latent class analysis revealed three preference patterns: (1) treatment effectiveness as the single most important attribute, (2) route of administration as the most important attribute, closely followed by frequency of use and psychological side effects and (3) severe side effects as the most important attribute followed by psychological side effects. In addition, disease duration and mild side effects influenced the patients’ choices.

**Conclusion:**

Respondents found either effectiveness, route of administration or severe side effects as the most important attribute. Patients noting effectiveness as most important were more willing than other patients to accept higher risks of side effects.

## Key points

- Patients with rheumatoid arthritis found either effectiveness of treatment, route of administration or probability of severe side effects to be the most important treatment attribute.
- Patients identifying effectiveness as most important were more willing than other patients to accept higher risks of side effects.
- This study could support shared decision-making by recognising the different preference patterns of patients.
- Results from this study have the potential to support regulatory marketing authorisations of rheumatoid arthritis treatments by revealing the minimum acceptable benefit levels required to compensate respondents for worsening levels of side effects.

## Introduction

Information about patient preferences has long been considered important for supporting patient-centeredness in clinical decisions [1]. Over the past decade, measuring patient preferences has evolved to use methods that quantify preferences in the clinical context [2–5]; these approaches have more recently also been used in rheumatology [6]. Recently, the interest in quantifying treatment preferences of patients with rheumatic diseases has been expanded to regulatory marketing authorisation decisions of new rheumatic disease medicines [7, 8]. Quantitative assessments of patient preferences may be important in regulatory marketing approvals in order to adjust decision-making to patient opinions on the meaning and significance of treatment attributes, such as the balance between estimated effects and adverse reactions [9, 10]. A better adjustment to patient preferences may also have a positive impact on patient adherence [9].

Patients with rheumatoid arthritis (RA) are often treated with multiple disease-modifying anti-rheumatic drugs (DMARD). DMARDs have different modes of action and characteristics, such as method and frequency of administration and probability of adverse events or monitoring requirements. Newly diagnosed patients with RA usually start with conventional synthetic DMARDs as first-line therapy. If first-line therapy is not tolerated or is ineffective, biologics or Janus kinases (JAK) inhibitors are recommended [11]. A potential advantage of JAK inhibitors is that they are given orally rather than subcutaneously or intravenously as is required for biologics [12].

Previous research has shown that cost, efficacy, and administration strongly influence patient preferences for second-line therapy—i.e., biologics or JAK inhibitors [2, 3, 13]. However, both biologics and JAK inhibitors are associated with side effects such as infections, increased blood and cholesterol levels, nausea, anxiety, and skin rash [12]. Therefore, clinicians should provide patients with specific information about treatments with these medicines, including the extent and probability of experiencing side effects. Although treatment costs can be an important determinant of preference, they are less relevant in countries with universal health care systems, as is the case for most of Europe.

Clinicians need to understand their patients’ preferences and perspectives when informing them about potential RA treatments so their patients can influence decisions about their treatment to align with their preferences [9, 10, 14]. Quantitative assessments of patient preferences have the potential to support both clinicians and regulators when they consider patient perspectives [7, 12]. Currently there is a lack of evidence about the extent to which patients feel that risks of side effects would be acceptable for new second-line treatments. This study assesses preferences regarding attributes of second-line treatments and heterogeneity within these preferences for patients with RA. These preferences are used to estimate the relative importance of different treatment characteristics and to calculate the minimum benefit levels patient require in order to accept higher levels of potential side effects.

## Methods

### Recruitment

Treatment preferences of patients with RA were assessed using a discrete choice experiment (DCE). An invitation to participate in the study was advertised to members of the Swedish Rheumatism Association via email, newspaper, newsletter, social media, mobile application, and the association’s website. The invitation to participate was also distributed to patients attending ten rheumatology clinics in Sweden and via an online research panel of patients with RA. A printed copy of the survey was distributed by the Rheumatology clinic at Uppsala University hospital. All participants received information about the study and provided their informed consent before completing the survey. The following inclusion criteria were used: established RA diagnosis, 18–80 years of age, and the ability to understand and answer the questions. Data were collected from November 2018 to August 2019. The survey was approved by the regional ethics review board in Uppsala, Sweden (Reg no. 2017/521, 2018/156). Data generation, storage and sharing were governed by the General Data Protection Regulation (GDPR) Act, Uppsala University data protection and security policies, and ethical consent provided.

### Methodology of discrete choice experiment

DCE, a cross-sectional survey method used to assess preferences, allows for quantitative assessment of patient preferences for health care policies, services, and interventions [15]. DCE, which uses random utility theory (RUT), aims to quantify the relative importance of one treatment characteristic over another treatment characteristic. RUT assumes that the value (utility) of a product can be determined by the value (utility) of the characteristics of that product (i.e., attributes) and their levels. Respondents in a DCE are presented with hypothetical scenarios (choice questions) with varying attributes and levels. Respondents are asked to choose their preferred option for each question [16]. The utility can be estimated by modelling the choices that respondents make between alternatives of treatments that are described by different choice questions [17]. DCEs can also be used to measure and explain heterogeneity within the preferences of patients [18].

### Attributes and levels

Using a step-wise approach, we identified attributes and levels for inclusion in the DCE. First, the analysis of a literature review of previous studies of patient preferences for DMARDs resulted in 12 potential treatment attributes [2, 3, 5, 14, 19–24]. Second, the attributes and levels identified in the literature review were discussed with a rheumatologist to make sure that they reflected current clinical practice. Third, three focus groups using the nominal group technique (NGT) were conducted with patients with RA (*n* = 7); these patients were asked to identify new attributes and rank all potential attributes from most to least important [25]. The focus groups were audio recorded, lasted for about 90 min, and conducted using an interview guide. Fourth, results from the focus groups were discussed during several validation meetings with one rheumatologist, the research team, and two patient research partners. These meetings revealed seven attributes: route of administration, frequency of use, probability of mild short-term side effects, probability of side effects changing appearance, probability of psychological side effects, probability of severe side effects, and effectiveness of treatment. Each attribute was revealed to have three levels based on current clinical knowledge of existing biologics and JAK inhibitors. Detailed information regarding the selection and description of the attributes and levels is available in the Supplementary material. All attributes and levels included in the DCE are displayed in Table 1.

**Table 1:** Attributes and levels

Attribute	Level 1	Level 2	Level 3
Route of administration	Tablet	Injection	Drip
Frequency of use	Daily	Weekly	Monthly
Probability of mild short-term side effects (nausea, vomiting or headache)	Common 1 in 10	Uncommon 1 in 100	Rare 1 in 1000
Probability of side effects changing appearance (hair loss, weight changes or skin rash)	Common 1 in 10	Uncommon 1 in 100	Rare 1 in 1000
Probability of psychological side effects (anxiety, mood changes, depression or sleep disturbance)	Common 1 in 10	Uncommon 1 in 100	Rare 1 in 1000
Probability of severe side effects that requires hospitalisation such as severe infections or allergic reactions	Common 1 in 10	Uncommon 1 in 100	Rare 1 in 1000
Effectiveness (the ability to decrease inflammation and swelling of the joints, also pain and other symptoms)	30% improvement. So out of 100 persons taking the treatment, 30 will get enough improvement; the rest will get a small or no improvement	50% improvement. So out of 100 persons taking the treatment, 50 will get enough improvement; the rest will get a small or no improvement	70% improvement. So out of 100 persons taking the treatment, 70 will get enough improvement; the rest will get a small or no improvement

### Experimental design and survey

**Fig. 1:**
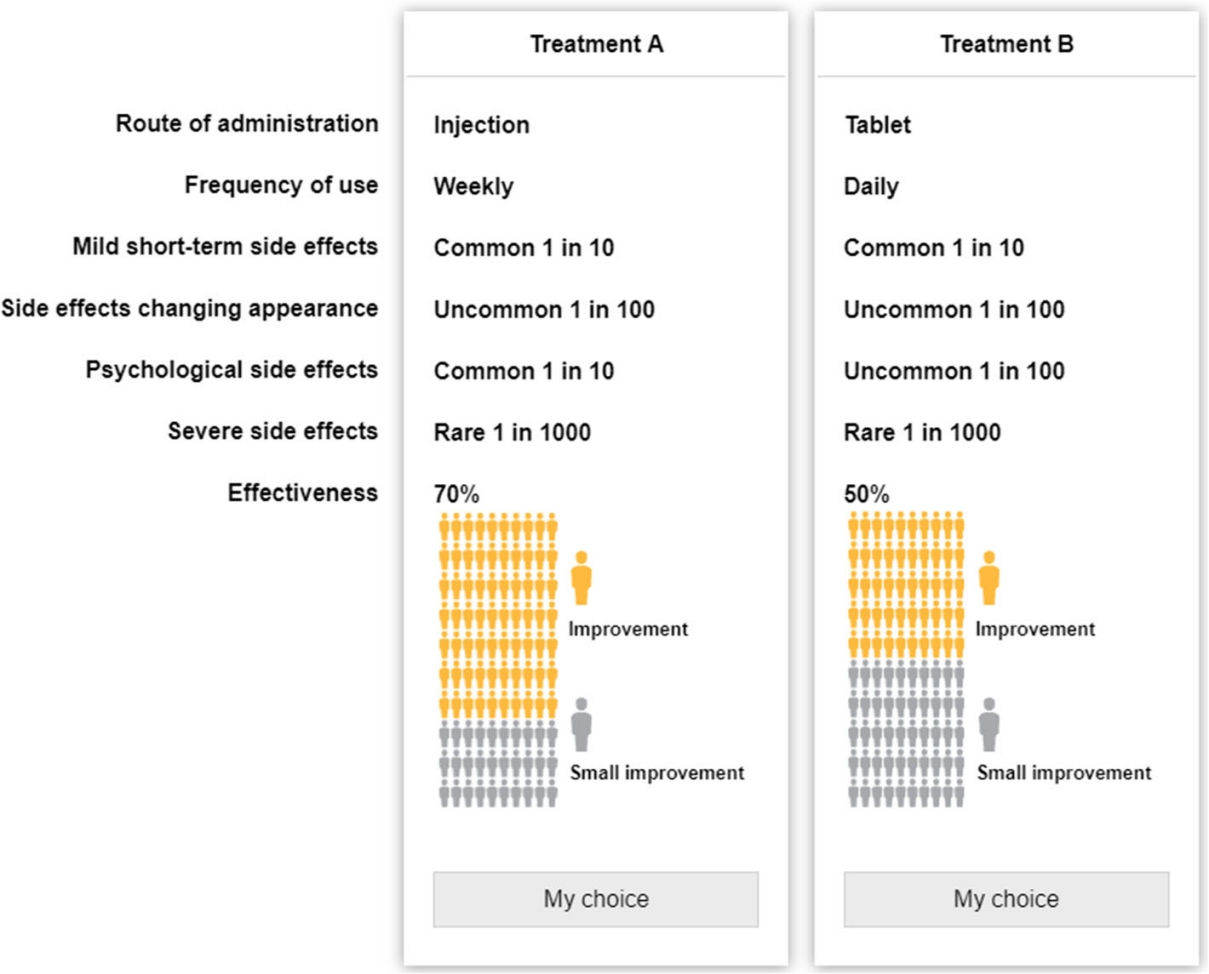
Example of a choice question

The survey started with information about RA and available treatment options before entering the DCE. The last section of the survey consisted of demographic and disease-related questions, health literacy [26], and numeracy [27]. The DCE had an attribute-based experimental design. Respondents were asked to choose their preferred treatment from two alternatives (see Fig. 1, example of a choice question). The choice questions also included a hover function with further explanations of the attributes and the levels (see Supplementary file for full text explanations of attributes and levels).

The survey was pilot tested with a subgroup (*n* = 22) of patients with RA and patient research partners. Six of the pilot tests were ‘think aloud’ interviews. The respondents were encouraged to articulate their thoughts while completing the survey. The language and the layout of the survey were slightly changed after the pilot test. Using the pilot test data, we fitted a multinomial logit (MNL) model and used the beta estimates as priors for the final experimental DCE design generated by NGene 1.0 (ChoiceMetrics, 2011), which is a *d* efficient (Bayesian) design [28]. A constraint was posed on the design: route of administration and frequency of use (e.g., if the route of administration was a tablet, the frequency of use could not be ‘monthly’). A total of 60 unique choice questions were divided into four blocks. Each respondent had to answer 15 unique choice questions. All attributes were displayed in each of the choice questions; three attributes were identical across the two offered alternatives to reduce the cognitive burden to respondents. We applied the decision-making scenario ‘think of yourself in a situation where your treatment is not working, your joints are swollen, you have pain or unbearable side effects and need to change to a second-line treatment’.

### Statistical analysis

SPSS® Statistics 20 and Nlogit® were used for analyses. Demographic data were analysed using descriptive statistics. Results were considered statistically significant if *P <* 0.05. The patients’ preferences were determined by attribute level estimates using a MNL model [29]. Latent class analysis (LCA) models were used for further analysis of the DCE data. Such models account for the multilevel structure of the data (i.e., every respondent answered multiple choice questions) and account for the investigation of preference heterogeneity. LCA models assume that there are two or more latent classes of data with different preferences. The classes are characterised by unobserved latent variables that can be related to a set of choice patterns. Once choice patterns have been stratified into classes, it is possible for the model to determine the probability that a respondent with certain characteristics will be assigned to each class [30]. The attributes were dummy coded (i.e., the mean effect for each attribute was normalised at zero) except for effectiveness that was effects-coded. The ‘likelihood ratio test’ and the Akaike information criterion (AIC) were used to determine the most appropriate model. A three-class model based on the utility is displayed below:

$$ {V}_{rta\mid c}={\beta}_{1\mid c}\ \mathrm{Route}\ {\mathrm{of}\ \mathrm{administration}}_{\mathrm{Tablet}\  rta\mid c}+{\beta}_{2\mid c}\ \mathrm{Route}\ {\mathrm{of}\ \mathrm{administration}}_{\mathrm{Injection}\  rta\mid c}+{\beta}_{3\mid c}\ \mathrm{Frequency}\ \mathrm{of}\ {\mathrm{use}}_{\mathrm{Daily}\  rta\mid c}+{\beta}_{4\mid c}\ \mathrm{Frequency}\ \mathrm{of}\ {\mathrm{use}}_{\mathrm{Weekly}\  rta\mid c}+{\beta}_{5\mid c}\ \mathrm{Mild}\ \mathrm{short}-\mathrm{term}\ {\mathrm{side}\ \mathrm{effects}}_{1\ \mathrm{in}\ 10\  rta\mid c}+{\beta}_{6\mid c}\ \mathrm{Mild}\ \mathrm{short}-\mathrm{term}\ {\mathrm{side}\ \mathrm{effects}}_{1\ \mathrm{in}\ 100\  rta\mid c}+{\beta}_{7\mid c}\ \mathrm{Appearance}\ {\mathrm{side}\ \mathrm{effects}}_{1\ \mathrm{in}\ 10\  rta\mid c}+{\beta}_{8\mid c}\ \mathrm{Appearance}\ {\mathrm{side}\ \mathrm{effects}}_{1\ \mathrm{in}\ 100\  rta\mid c}+{\beta}_{9\mid c}\ \mathrm{Psychological}\ {\mathrm{side}\ \mathrm{effects}}_{1\ \mathrm{in}\ 10\  rta\mid c}+{\beta}_{10\mid c}\ \mathrm{Psychological}\ {\mathrm{side}\ \mathrm{effects}}_{1\ \mathrm{in}\ 100\  rta\mid c}+{\beta}_{11\mid c}\ \mathrm{Severe}\ {\mathrm{side}\ \mathrm{effects}}_{1\ \mathrm{in}\ 10\  rta\mid c}+{\beta}_{12\mid c}\ \mathrm{Severe}\ {\mathrm{side}\ \mathrm{effects}}_{1\ \mathrm{in}\ 100\  rta\mid c}+{\beta}_{13\mid c}\ {\mathrm{Effectiveness}}_{rta\mid c} $$

The utility component (*V*) describes the utility that respondent ‘*r*’ belonging to class ‘*c*’ reported for alternative ‘*a*’ in choice question ‘*t*’. The attribute level estimates of each attribute level are represented by *β*_1_–*β*_13_. A class assignment model was fitted after the specified utility function. Several demographic and disease-related variables were tested for their potential impact on class membership in the LCA: age, gender, numeracy, health literacy, education level, disease duration, occupational status, and experience with DMARD treatment and side effects. The final class assignment utility function was:

$$ {V}_{\mathrm{rc}}={\beta}_{1\mid c}\ {\mathrm{disease}\ \mathrm{duration}}_r+{\beta}_{2\mid c}\ \mathrm{and}\ \mathrm{mild}\ {\mathrm{side}\ \mathrm{effects}}_r $$

A significant attribute estimate within a certain class indicates that this attribute contributes to the decision-making process of respondents who belong to that class. The sign of the beta indicates whether the attribute level has a positive or negative effect on the utility.

To calculate the relative importance of the attributes, the difference between the highest and lowest estimates of the attribute level was calculated for each attribute. The largest difference value was given a 1, representing the attribute that was deemed most important by respondents. The other difference values were divided by the largest difference value, resulting in a relative distance between all other attributes and the most important attribute.

A minimum acceptable benefit (MAB) for changes in attribute levels was calculated. The MAB is interpreted as the minimum change in effectiveness that respondents would require (on average) to accept changes to a less desirable level in another attribute (probability of getting a certain side effect by 10%, 1%, and 0.1%). MAB was estimated as the difference between the preference weights (parameters) for two levels ‘*l*’ of an attribute divided by the preference weight, *β*_*k =* effectiveness_, which is the unit change in the level of benefit:

$$ \mathrm{MAB}=-\frac{\left({\beta}_{k,l=2}-{\beta}_{k,l=1}\right)\ }{\left({\beta}_{k=\mathrm{effectiveness},}\right)} $$

## Results

### Respondents

In total, 422 patients completed the full survey although 29 were removed after testing for flat-lining (choosing option A at least 13 out of 15 times) and 35 were removed because they answered the survey in under 5 min. Most of the respondents were female (77%). The respondents represented all age categories between 18 and 80 years of age. The level of education was categorised into low (*n* = 105), medium (*n* = 86), or high (*n* = 162). A full overview of patient and disease characteristics is presented in Table 2.

**Table 2:** Patient and disease characteristics

Item	***N***	***N*** in %
**Total**	**358**	100
Gender		
Female	272	77
Male	83	23
Age		
18–24	15	4
25–34	42	12
35–44	31	9
45–54	64	18
55–64	99	28
65–80	105	30
Education level		
Low (elementary school, primary school, real school or similar, 2-year high school or vocational school, 3–4 year high school)	105	30
Medium (college or university shorter than 3 years)	89	25
High (college or university 3 years or longer)	162	45
Occupational status		
Full time employee, part time employee, parental leave/occupational leave	154	43
Work part time since RA, long-term sick leave, sick pension	79	22
Age pensioner/unemployed	177	33
Other	6	2
Health literacy		
Sufficient	197	55
Problematic	134	38
Lacking	24	7
Numeracy		
High	28	8
Medium	212	60
Low	113	32
Disease duration		
1–12 months	22	6
1–5 years	88	25
5–10 years	67	19
More than 10 years	179	50
Time till onset of drug effect		
0–3 months	121	34
3–12 months	87	25
1–2 years	33	9
2–5 years	37	11
More than 5 years	32	9
Still not working	43	12
Experience with treatment		
First line treatment only (csDMARDs)	182	51
Second line treatment, biologics	116	32
JAK inhibitors	12	3
Experience with side effects		
Mild short term	205	57
Appearance	154	43
Psychological	137	38
Severe	80	22
No side effects	89	24

### Preferences and relative importance

The multinomial logit model revealed that all of the attribute estimates significantly contributed to the decision-making process of respondents. The sign of the beta indicates whether the attribute level has a positive or negative effect on the utility. On average, respondents preferred a tablet and injection over a drip (as indicated by the positive estimates for tablet and injection). The respondents also preferred monthly over weekly or daily medication. A strong disutility for the highest frequency of side effects was found in all classes. Finally, respondents preferred the medicine with the highest effectiveness. The directions of the effects of the attributes on utility were as expected, which confirms that respondents understood the choice questions. On average, the most important attribute for respondents was the probability of severe side effects. Treatment effectiveness was the second most important attribute, closely followed by the probability of psychological side effects. Route of administration came in fourth place followed by frequency of use, probability of mild short-term side effects, and side effects changing appearance.

### Preference heterogeneity

Considerable heterogeneity was found in the preferences, as can be seen in the three classes representing differences in preferences in Table 3. The average probability of respondents belonging to one of the classes was 34%, 28%, and 38%, respectively. The model fit significantly improved when including disease duration and experience of mild short-term side effects (loglikelihod = − 2495 and − 2491, *P* < 0.05) to the class assignment model.

**Table 3:** Preferences of patients based on latent class analysis

	Class 1 estimate	SE	RI	Class 2 estimate	SE	RI	Class 3 estimate	SE	RI
Route of administration									
Tablet	1.22***	0.27	0.25	0.92***	0.20	1.00	1.14***	0.19	0.31
Injection	0.37**	0.17		0.51***	0.15		0.64***	0.16	
Drip (ref)									
Frequency of use									
1 a day	− 1.00***	0.18	0.22	− 0.75***	0.16	0.82	− 0.59***	0.14	0.16
1 a week	− 0.47***	0.17		− 0.23	0.14		− 0.02	0.16	
1 a month (ref)									
Probability of mild short-term side effects									
1 in 10	− 0.30*	0.17	0.06	− 0.27*	0.14	0.29	− 0.44**	0.17	0.12
1 in 100	− 0.15	0.13		− 0.08	0.12		− 0.06	0.14	
1 in 1000 (ref)									
Probability of side effects changing appearance									
1 in 10	− 0.87***	0.20	0.18	− 0.34**	0.16	0.11	− 1.55***	0.21	0.42
1 in 100	− 0.04	0.17		− 0.10	0.14		− 0.48***	0.15	
1 in 1000 (ref)									
Probability of psychological side effects									
1 in 10	− 1.11***	0.23	0.23	− 0.75***	0.18	0.82	− 2.61***	0.28	0.72
1 in 100	− 0.01	0.18		− 0.62***	0.15		− 0.34**	0.17	
1 in 1000 (ref)									
Probability of severe side effects									
1 in 10	− 1.75***	0.27	0.36	− 0.21	0.18	0.23	− 3.65***	0.39	1.00
1 in 100	− 0.82***	0.16		− 0.08	0.12		− 0.79***	0.16	
1 in 1000 (ref)									
Effectiveness (linear)	0.12***	0.01	1.00	0.01**	0.00	0.43	0.04***	0.00	0.44
**Class probability model**									
Constant	1.32	0.96		2.51***	0.96		–	–	
Disease duration	− 0.16	0.12		− 0.32***	0.12		–	–	
Experience with mild side effects	− 0.46	0.36		− 0.99**	0.39		–	–	
Average class probability	0.34			0.28			0.38		

“***,” “**” and “*” indicate significance at 1%, 5%, and 10%, respectively. *RI* relative importance

**Fig. 2:**
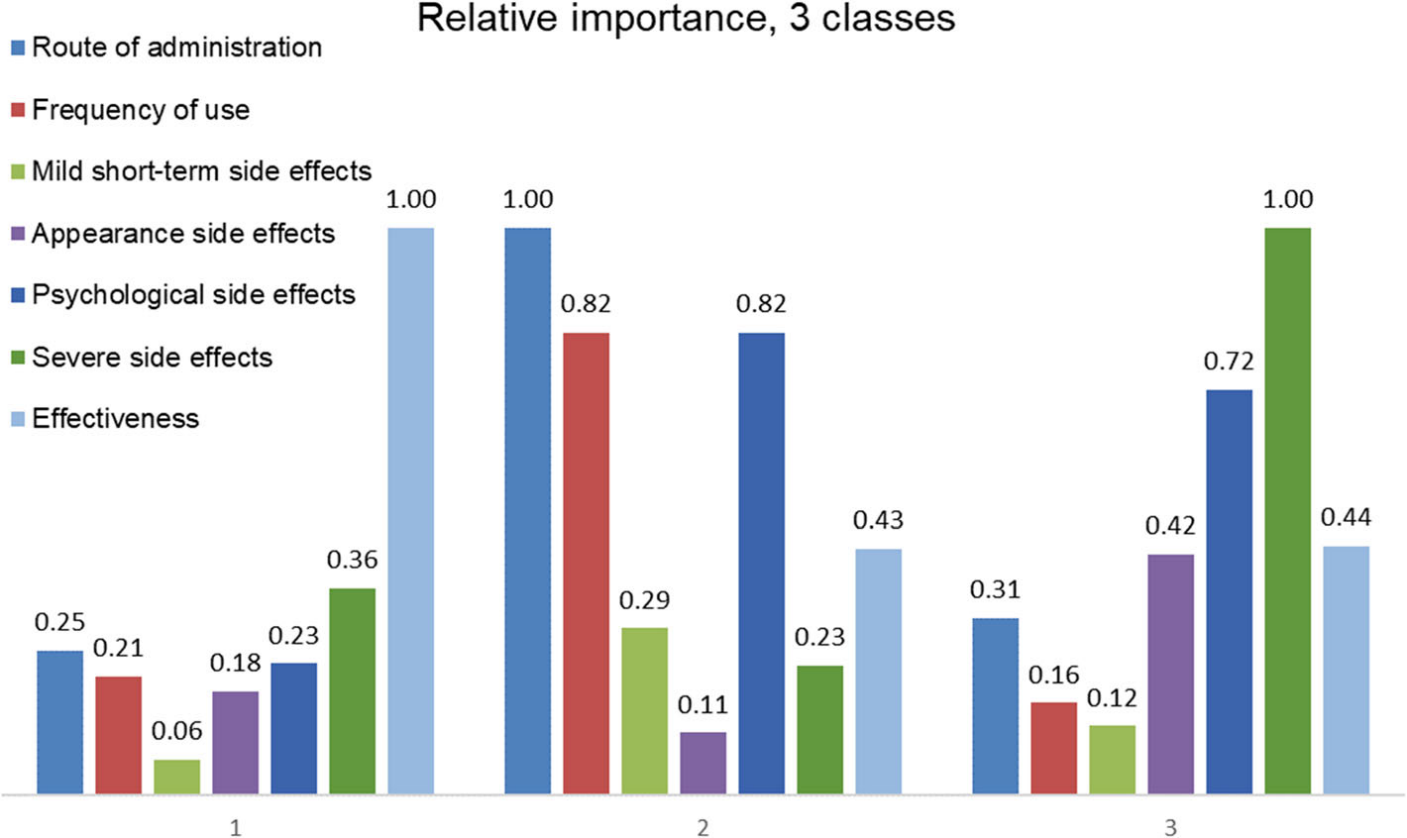
Relative importance score of attributes

Although the directions of the impact of the attribute levels on utility were the same in all classes, high levels of heterogeneity were observed with respect to the importance of the attribute levels. The relative importance (RI) score of the attributes was separately calculated for the three classes of the latent class analysis (Fig. 1). According to class 1, treatment effectiveness was the single most important attribute. While in class 2, route of administration was the most important attribute, closely followed by frequency of use and psychological side effects. Severe side effects were the most important attribute followed by psychological side effects for class 3. Respondents with newly diagnosed RA and no experiences of mild short-term side effects were more likely to belong to class 2, whereas respondents with longer disease duration and previous mild short-term side effects were more likely to belong to class 3 (Fig. 2).

### Minimum acceptable benefit

Table 4 shows the minimum acceptable benefit (MAB) levels required (in percentage point increases in effectiveness) to compensate respondents for worsening levels of probability of certain side effects. Due to preference heterogeneity, large differences were found in the MAB across the three classes. In class 1, only a small benefit was needed to accept a switch to a less favourable frequency of side effects. In the other two classes, respondents would require a larger increase in effectiveness to accept an increase in risk of side effects. The highest MAB levels were seen in class 3 respondents for moving from a 0.1% probability of severe side effects to a 10% probability, which required a 91.3 percentage point increase in treatment effectiveness. The second highest MAB level was seen in class 2 for moving from a 0.1% probability of psychological side effects to a 10% probability, which required a 75.0 percentage point increase in treatment effectiveness.

**Table 4:** Minimum acceptable benefit for changes in attribute levels

Attribute	Change	Minimum acceptable benefit in percentage		
		Class 1	Class 2	Class 3
Probability of mild short-term side effects	Moving from 0.1 to 10%	2.5	27.0	11.0
	Moving from 0.1 to 1%	1.3	–	1.5
	Moving from 1 to 10%	1.3	35.0	9.5
Probability of side effects changing appearance	Moving from 0.1 to 10%	7.3	34.1	38.8
	Moving from 0.1 to 1%	–	10.0	12.0
	Moving from 1 to 10%	7.6	24.0	26.8
Probability of psychological side effects	Moving from 0.1 to 10%	9.3	75.0	65.3
	Moving from 0.1 to 1%	–	62.0	8.5
	Moving from 1 to 10%	9.3	13.0	56.8
Probability of severe side effects	Moving from 0.1 to 10%	14.6	21.0	91.3
	Moving from 0.1 to 1%	–	8.0	18.8
	Moving from 1 to 10%	21.4	13.0	71.5

## Discussion

This study assesses preferences regarding second-line treatment and heterogeneity within these preferences among patients with RA as well as estimates of the relative importance of different treatment characteristics. In addition, this study calculates the minimum benefit levels patients require in order to accept higher levels of potential side effects. Respondents found either effectiveness of treatment, route of administration, or probability of severe side effects to be most important. This study also reveals that disease duration and experience with mild side effects had an impact on patients’ choices. For newly diagnosed patients with no experience of mild side effects, route of administration (with oral administration being most preferred) was the most important treatment attribute. This preference might be due to wanting a treatment that fits with current lifestyle, since taking a tablet is less invasive and more convenient than a self-administered injection or having an infusion [23]. In addition, respondents might find it easier to understand the impact of route of administration on daily life, whereas relatively small changes in side effects may be more complicated to understand.

Findings from this study are in line with previous research reporting on different patterns of preferences of patients with RA, as the importance of effectiveness and severe side effects [2, 3, 13]. However, the attributes and levels for this study address more side effects in the choice questions, such as the probability of psychological side effects or side effects changing appearance and the probabilities of these side effects.

For patients whose choices were most influenced by treatment effectiveness, the impact of side effects on decision-making was marginal. These patients might be recognised as a subgroup with increased willingness to accept higher risk of side effects for an increase in effectiveness. Such patients might be willing to try a new orally administered treatment even though there is uncertainty regarding long-term safety outcomes. Newly diagnosed patients preferred an oral medication over all other attributes; however, they did not accept an increased risk of severe side effects. Similarly, patients with longer disease duration and experience with mild side effects were less willing to accept a treatment with a higher risk of severe side effects.

Previous studies have revealed that rheumatologists and patients with RA have different treatment preferences [14, 31]. This study could support rheumatologists and patients in shared decision-making by identifying which attributes should be the focus of treatment discussions. This study has also revealed the trade-offs that patients with RA are willing to make, a finding that may help patients recognise what is most important from an individual perspective. Tailoring treatment according to patients’ preferences may increase treatment satisfaction and compliance, which could improve treatment outcomes in patients with RA [3].

For regulatory decision-making, considering preference heterogeneity in marketing authorisations or in post authorisations may lead to decisions that are more acceptable to the end users. Treatment satisfaction may increase for patients with a higher acceptance of side effects if the prospect of effectiveness is higher [32, 33].

There are some limitations of this study. First, several sources were used to recruit patients with RA and there was limited control over patient selection, it was not possible to calculate the response rate. However, respondents were only able to participate at one time and no duplicates were found in the patient sample. The patient characteristics (age, gender, education and treatment experience) suggest a representative sample of the Swedish RA population [34]. This article provides a useful addition to the literature by assessing the Swedish population. The results may not be generalizable to other European countries as the health care systems are different. However, there is some concordance between the results of similar, previous studies in a range of countries [35].

Future research should focus on other important disease-related characteristics such as disease activity and risk propensity, characteristics that may influence respondents’ preferences. Research needs to develop methods and guidelines to bring in the results of patient preference assessments in both regulatory marketing approval decisions and in the clinical context of shared decision-making.

## Conclusions

Respondents’ choices were most influenced by effectiveness of treatment, route of administration or probability of severe side effects. Patients who found effectiveness of treatment to be most important only reported a marginal impact of side effects; these patients might be recognised as a subgroup of patients more willing to accept higher risk of side effects for increased effectiveness. Other patients may not accept a switch associated with increased risk of severe side effects. This study could support personalisation of treatment with second-line treatment by recognising the different preference patterns of patients and the minimum acceptable levels of benefit. Consideration of preference heterogeneity in marketing authorisations or in post authorisations may lead to decisions more acceptable to the end users.

## Data Availability

The datasets during and/or analysed during the current study available from the corresponding author on reasonable request.
